# Impact of joint management of a COVID-19 mother and her newborn on the virus transmission: a case report

**DOI:** 10.1186/s12985-021-01598-w

**Published:** 2021-06-28

**Authors:** Maria Chiara De Nardo, Anna Rita Bellomo, Francesca Perfetti, Francesco Antonino Battaglia, Miriam Lichtner, Riccardo Lubrano

**Affiliations:** 1 Pediatric and Neonatal Unit, Sapienza University of Rome, Polo Pontino, Santa Maria Goretti Hospital, Via A. Canova, 04100 Latina, Italy; 2 Gynecological and Obstetrical Unit, Santa Maria Goretti Hospital, Latina, Italy; 3 Infectious Diseases Unit, Sapienza University of Rome, Polo Pontino, Santa Maria Goretti Hospital, Latina, Italy

**Keywords:** SARS-CoV2 transmission, Neonates, Breastfeeding, Rooming-in

## Abstract

**Background:**

Since last year, COVID-19, the disease caused by the novel Sars-Cov-2 virus, has been globally spread to all the world. COVID-19 infection among pregnant women has been described. However, transplacental transmission of Sars-Cov-2 virus from infected mother to the newborn is not yet established. The appropriate management of infants born to mothers with confirmed or suspected COVID-19 and the start of early breastfeeding are being debated.

**Case presentation:**

We report a case of the joint management of a healthy neonate with his mother tested positive for Covid-19 before the delivery and throughout neonatal follow-up. The infection transmission from the mother to her baby is not described, even after a long period of contact between them and breastfeeding.

**Conclusion:**

It may consider an appropriate practice to keep mother and her newborn infant together in order to facilitate their contact and to encourage breastfeeding, although integration with infection prevention measures is needed.

## Background

Since COVID-19 disease spread, multiple reports described the novel SARS-CoV-2 infection among pregnant women. [1] According to neonatologists, the major issue to address is the establishment of a correct management of COVID-19 mothers and her infants after childbirth as well as the safety of early breastfeeding.

Data from current literature suggest no difference in risk of neonatal SARS-CoV-2 infection whether the neonate is cared for in a separate room or remains in the room of his mother with suspected or confirmed SARS-CoV-2 infection [2–6]. Overall, the neonate’s risk for acquiring SARS-CoV-2 from its mother is low, however, mothers should wear a mask and practice hand hygiene during contact with their infants in order to minimize the risk of viral transmission. The findings of a prospective, multicenter study enrolling mothers with SARS-CoV-2 infection who were eligible for rooming-in practice based on their clinical condition, provide evidence-based information on the management of mother-infant dyads in case of SARS-CoV-2 maternal infection. The authors suggested that rooming-in and breastfeeding can be practiced in women who are able to care for their infants as the risk of transmission was very low [6].

The risk of SARS-CoV-2 transmission from ingestion of breast milk is unclear. Although small series described that all samples of breast milk from mothers with COVID-19 tested negative [7, 8], other authors identified samples of breast milk positive for SARS-CoV-2 by RT-PCR [9–12]. However, samples that are SARS-CoV-2 RT-PCR positive do not necessarily contain replication-competent virus [12]. There is general consensus that breastfeeding should be encouraged.

We present a case report of the joint management of a COVID-19 mother and her baby aiming to promote their interaction and to support breastfeeding.

## Case presentation

At term of gestation, a 36-year-old woman presented at the Labour Unit of our hospital. Twenty days before, she had fever and cough and was identified as SARS-CoV-2 positive. Since then, the patient was asymptomatic. In the family history, her parents also tested positive for COVID-19. On the admission to the hospital, the patient was put a surgical mask and nasopharyngeal and oropharyngeal swabs were performed. The samples were tested for SARS-CoV-2 by using Allplex™ 2019-nCoV Assay, which is multiplex Real-Time PCR (RT-PCR) assay for simultaneous detection of 3 target genes of SARS-CoV-2 (RdRP, N and E gene) in a single tube. The result of RT-PCR-RNA test confirmed woman’s infection with two genes being detected (RdRP and N). The day after, a 3290 g male infant was born by vaginal delivery at 40 weeks of gestation, with Apgar scores of 9 and 10. Skin to skin contact was performed in labour room.Amniotic fluid samples,vaginal and placenta swab specimens obtained were tested negative for SARS CoV-2 by RT-PCR-RNA test. After delivery, the infant was moved to room-in with the mother. The baby's cradle was placed at a distance of 2 m from the mother's head. Breastfeeding was immediately commenced to benefit of the antiviral potential of colostrum and subsequently of breast milk. During breastfeeding, the mother was indicated to wear the surgical face mask and to follow hygienic rules (hand washing) in order to avoid viral transmission by droplet, according to the Neonatal Italian Society guidelines [1]. Breast milk samples, collected via pumps into sterile containers 10 min after baby feeding and following meticulous nipple hygiene with chlorhexidine 2%, tested negative for SARS-CoV-2 by using Allplex™ 2019-nCoV Assay. Nasopharyngeal and oropharyngeal swabs obtained to detect SARS-COV2 on the infant at 6, 24 and 48 h of age were all negative whereas on the same timings the mother still resulted positive for COVID-19. Blood tests of the baby were unremarkable. On day 2 the baby was discharged home with his mother. At 28-day follow-up the baby was in a good condition. Breastfeeding, integrated with strict hygiene measures, was not discontinued at home although mother was still SARS-CoV 2 positive. Repeated nasopharyngeal and oropharyngeal swabs on the infant at 4, 14, 18 and 28 days of age were all negative, whereas on the same days the mother still tested positive for COVID-19. In all the nasopharyngeal swabs obtained from the mother, the cycle threshold (Ct) value of RT PCR targeting N gene and RdRPgene, was between 33 and 38.7.

## Discussion

The appropriate management recommendations of neonates born to COVID-19 woman was largely unclear at the beginning of pandemic events.

Authors and Initial American Academy of Pediatrics (AAP) guidance [13, 14], published when our case was reported, recommended temporary separation of newborns from infected mothers as the safest means to prevent the newborn infant from becoming infected. This cautious guidance was provided because the risks of perinatally and postnatally-acquired newborn infection were unknown. After months of national and international experience with newborns born to mothers who have tested positive for SARS-CoV-2, AAP [15] stated that the likelihood that an infant has a positive PCR test for SARS-CoV-2 is similar for infants who are separated from their mothers and for infants who room-in with mothers using infection prevention measures.

On the contrary, since COVID-19 disease spread, The Italian Society of Neonatology, World Health Organization (WHO), United Nations Children’s Fund (UNICEF) and the Royal College of Obstetricians & Gynecologists [16–19] supported the joint management of infant and mother, if she is in good health condition, using the recommended hygiene procedures to prevent transmission of the viral infection with respiratory secretions. In our case we followed that management guidance without infection transmission from mother to neonate. Regarding breastfeeding, available studies report no evidence of SARS-CoV-2 in human breast milk. However, in a single caseGroß et al. detected SARS-CoV-2 RNA in breast milk samples from a mother with mild COVID-19 symptoms, whose baby also tested positive for SARS-CoV-2. No definite conclusions were drawn by the authors whether the child was infected through breastfeeding or other modalities of transmission. They stated that further studies of milk samples are needed to establish whether breast milk could be a possible vehicle of virus transmission [11].

Other authors, analyzing 64 milk samples collected from symptomatic and/or asymptomatic mothers with COVID-19, observed one breast milk sample with detectable SARS-CoV-2 RNA. However, the viral culture for all the samples was negative, including the sample that tested positive for viral RNA. Authors concluded that SARS-CoV-2 RNA does not represent replication-competent virus and that breast milk may not be a source of infection for the infant [12]. The AAP and Centers for Disease Control and Prevention (CDC) [15, 20] strongly supports breastfeeding as the best choice for infant feeding as the current evidence suggests that breast milk may not be considered source of infection when a woman, previously identified as positive for SARS-CoV-2, is asymptomatic or pauci-symptomatic and applies the preventive infection transmission strategies.

This approach considers the well-known mother-infant benefits of breastfeeding, the low likelihood of maternal infection transmission to the newborn when infection control measures are taken, and the mild symptoms of newborn infection when it occurs. An observational study from New York City that tested and followed 82 infants of 116 mothers who tested positive for SARS-CoV-2, supports this policy. The authors observed that no infants tested positive for SARS-CoV-2 postnatally, although rooming-in and breastfeeding were practiced using infection precautions (appropriate hand hygiene, breast cleansing, and placement of a surgical mask) [21].

**Fig. 1:**
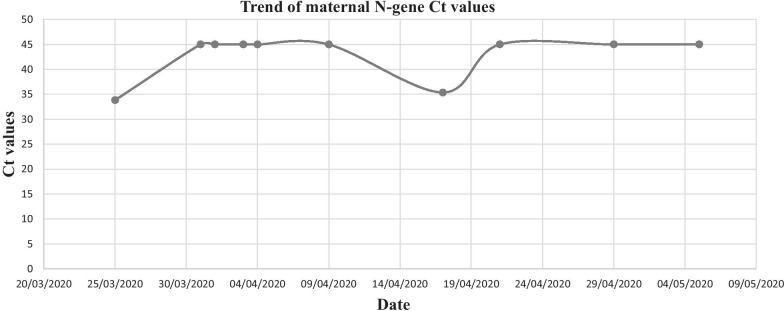
Trend of maternal N-gene Ct values during time of infection

In our case, infection transmission from mother to infant is not described, even after a long period of contact between them and breastfeeding. This may be explained also by a presumptive maternal low viral load indicated by high Ct in NP swabs (as shown in Fig. 1), according to La Scola et al. who observed that patients with COVID-19 with Ct above 33–34 were not contagious. Furthermore, a significant inversely proportional relationship between Ct value and culture positivity rate was reported [22]. To date it is still debated whether CT values correlate with COVID-19 severity. Among general population, it has been explored the use of CT values as a marker to predict degree of clinical severity and patient’s contagiousness. Some authors observed that higher viral load significantly associated with disease severity [23, 24], and others found no correlation [25–27]. Identifying a biomarker of potential infection transmission and disease severity may be particularly interesting in risk stratifying COVID-19 mothers. Our case suggests that the analysis of Ct value in maternal NP swabs could be helpful to monitor viral load and reassure clinicians that the mother may have not been infectious. However, as we reported only a single case, our speculations should be considered cautiously.

## Conclusion

To conclude, we may consider an appropriate practice to keep mother and her newborn infant together in order to facilitate their contact and to encourage breastfeeding, although integration with infection control measures is needed. Additional well-designed multicentric studies and metanalysis are advocated to further elucidate which is the correct clinical practice to apply.

## Data Availability

Data are available from the corresponding author on reasonable request.

## Abbreviations

COVID-19: Coronavirus disease 2019
SARS-CoV-2: Severe acute respiratory syndrome coronavirus 2
PCR: Polymerase chain reaction
RT-PCR: Real-time polymerase chain reaction
Ct: Cycle threshold
AAP: American Academy of Pediatrics
WHO: World Health Organization
UNICEF: United Nations Children's Fund
CDC: Centers for Disease Control and Prevention

## References

[1] Chen H, Guo J, Wang C, Luo F, Yu X, Zhang W (2020). Clinical characteristics and intrauterine vertical transmission potential of COVID-19 infection in nine pregnant women: a retrospective review of medical records. Lancet.

[2] Walker KF, O'Donoghue K, Grace N (2020). Maternal transmission of SARS-COV-2 to the neonate, and possible routes for such transmission: a systematic review and critical analysis. BJOG.

[3] CDC. Care for newborns. https://www.cdc.gov/coronavirus/2019-ncov/hcp/caring-for-newborns.html. Accessed 13 Nov 2020.

[4] Cojocaru L, Crimmins S, Sundararajan S (2020). An initiative to evaluate the safety of maternal bonding in patients with SARS-CoV-2 infection. J Matern Fetal Neonatal Med.

[5] Gale C, Quigley MA, Placzek A (2021). Characteristics and outcomes of neonatal SARS-CoV-2 infection in the UK: a prospective national cohort study using active surveillance. Lancet Child Adolesc Health.

[6] Ronchi A, Pietrasanta C, Zavattoni M (2021). Evaluation of rooming-in practice for neonates born to mothers with severe acute respiratory syndrome coronavirus 2 infection in Italy. JAMA Pediatr.

[7] Elshafeey F, Magdi R, Hindi N (2020). A systematic scoping review of COVID-19 during pregnancy and childbirth. Int J Gynaecol Obstet.

[8] Liu W, Wang J, Li W (2020). Clinical characteristics of 19 neonates born to mothers with COVID-19. Front Med.

[9] Kirtsman M, Diambomba Y, Poutanen SM (2020). Probable congenital SARS-CoV-2 infection in a neonate born to a woman with active SARS-CoV-2 infection. CMAJ.

[10] Wu Y, Liu C, Dong L (2020). Coronavirus disease 2019 among pregnant Chinese women: case series data on the safety of vaginal birth and breastfeeding. BJOG.

[11] Groß R, Conzelmann C, Müller JA (2020). Detection of SARS-CoV-2 in human breastmilk. Lancet.

[12] Chambers C, Krogstad P, Bertrand K (2020). Evaluation for SARS-CoV-2 in breast milk from 18 infected women. JAMA.

[13] Wang L, Shi Y, Xiao T, Fu J, Feng X, Mu D (2020). Chinese expert consensus on the perinatal and neonatal management for the prevention and control of the 2019 novel coronavirus infection (first edition). Ann Transl Med.

[14] Puopolo K, Kimberlin D (2020). Management of infants born to mothers with COVID-19 date of document: April 2, 2020. Am AcadPediatr Comm Fetus Newborn Sect Neonatal Perinat MedComm Infect Dis.

[15] Management of infants born to mothers with suspected or confirmed COVID-19 date of document: November 2, 2020. Am Acad Pediatr Comm Fetus Newborn, Sect Neonatal Perinat Med Comm Infect Dis. 2020.

[16] Davanzo R, Moro G, Sandri F, Agosti M, Moretti C, Mosca F (2020). Breastfeeding and coronavirus disease-2019. Ad interim indications of the Italian Society of Neonatology endorsed by the Union Of European Neonatal & Perinatal Societies. Matern Child Nutr..

[17] WHO. Clinical Management Of Severe Acute Respiratory Infection (SARI) When COVID-19 Disease Is Suspected: Interim Guidance V 1.2. WHO; 2020.

[18] https://www.unicef.org/stories/novel-coronavirus-outbreak-what-parents-should-know. Accessed 24 March 2020.

[19] Royal College Of Obstetricians And Gynaecologists. Coronavirus (COVID-19) Infection In Pregnancy Information For Healthcare Professionals. Centers For Disease Control And Prevention. 2020

[20] https://www.cdc.gov/coronavirus/2019-ncov/prepare/prevention.html. Accessed 20 May 2020.

[21] Salvatore CM, Han JY, Acker KP (2020). Neonatal management and outcomes during the COVID-19 pandemic: an observation cohort study. Lancet Child Adolesc Health.

[22] La Scola B, Le Bideau M, Andreani J (2020). Viral RNA load as determined by cell culture as a management tool for discharge of SARS-CoV-2 patients from infectious disease wards. Eur J Clin Microbiol Infect Dis.

[23] Zheng S, Fan J, Yu F (2020). Viral load dynamics and disease severity in patients infected with SARS-CoV-2 in Zhejiang province, China, January-March 2020: retrospective cohort study. BMJ..

[24] Liu Y, Yan LM, Wan L (2020). Viral dynamics in mild and severe cases of COVID-19. Lancet Infect Dis.

[25] He X, Lau EHY, Wu P (2020). Temporal dynamics in viral shedding and transmissibility of COVID-19. Nat Med.

[26] To KK, Tsang OT, Leung WS (2020). Temporal profiles of viral load in posterior oropharyngeal saliva samples and serum antibody responses during infection by SARS-CoV-2: an observational cohort study. Lancet Infect Dis.

[27] Huang JT, Ran RX, Lv ZH (2020). Chronological changes of viral shedding in adult inpatients with COVID-19 in Wuhan. China. Clin Infect Dis.

